# Virologic and Immunologic Characterization of COVID-19 Recrudescence after Nirmatrelvir/Ritonavir Treatment

**DOI:** 10.21203/rs.3.rs-1662783/v1

**Published:** 2022-05-18

**Authors:** Aaron F. Carlin, Alex E. Clark, Antoine Chaillon, Aaron F. Garretson, William Bray, Magali Porrachi, AsherLev T. Santos, Davey M. Smith

**Affiliations:** UC San Diego; UC San Diego; UC San Diego; UC San Diego; UC San Diego; UC San Diego; California State University San Marcos; UC San Diego

**Keywords:** COVID-19, Recrudescence, Nirmatrelvir, resistance, antibody

## Abstract

We isolated a SARS-CoV-2 BA.2 variant from a person with COVID-19 recrudescence after nirmatrelvir/ritonavir treatment. Antiviral sensitivity and neutralizing antibody testing was performed and compared with parental SARS-CoV-2 and multiple variants of concern. We found that neither NM resistance nor absence of neutralizing immunity were likely causes of the recrudescence.

Early administration of the oral protease inhibitor, Nirmatrelvir, combined with ritonavir (NM/r) (Paxlovid^™^) can reduce severe disease due to COVID-19^1^, but virologic and symptomatic rebound after NM/r treatment was recently reported^2^. We evaluated if NM resistance or impaired humoral immunity contributed to a case of COVID-19 recrudescence after NM/r treatment.

Three of four boosted adult travelers acquired COVID-19 after returning to the United States from South Africa and were treated with NM/r. All cases resolved quickly except one with rebounding symptoms associated with high viral shedding and culturable virus 5 days after an NM/r course. We isolated a SARS-CoV-2 pango lineage BA.2 virus (PRSD01) from a nasopharyngeal swab following development of worsening symptoms. SARS-CoV-2 full genome sequences isolated from the nasopharyngeal swab sample and culture isolate were assigned as SARS-CoV2 BA.2 lineage. Sequence comparison to the BA.2 reference showed no amino acid difference in any coding region, including ORF1a and Spike protein. Phenotypic analysis of the antiviral susceptibility of BA.2 PRSD01 (isolate), WA1/2020 (parental), B.1.617.2 (delta), BA.1 and BA.2.3 variants to NM and remdesivir were conducted. The half maximal inhibitory concentrations (IC50) of NM against BA.2 PRSD01 were 2.0, 1.8, 1.7, and 2.0 fold lower than the parental, delta, BA.1 and BA.2.3 strains respectively (Figure 1A). As a control, we determined the IC50 for remdesivir, a drug to which this individual was not exposed, for each strain. The remdesivir IC50 for BA.2 PRSD01 was 2.0, 1.8, 1.1, and 1.3 higher than the parental, delta, BA.1 and BA.2.3 strains respectively (Figure 1A).

We next evaluated the susceptibility of the viral panel to the neutralizing antibody response in the plasma from the patient and two controls. Both controls were fully vaccinated and boosted with the BNT162b2 mRNA vaccine (Pfizer–BioNTech), but one had been infected with and recovered from a SARS-CoV-2 infection with symptom onset 3 days prior to the individual who experienced NM/r recrudescence. The average half maximal authentic virus neutralizing antibody concentrations (NT50) against BA.2 PRSD01, BA.1, and parental were, 1668, 1170 and 5239 respectively in the NM/r treated patient. These levels were 8.9, 7.1 and 2.1 times higher than the boosted control but 2.0, 1.7 and 2.6 times lower than the boosted and infected control (Figure 1B).

This study found that neither development of NM resistance or absence of neutralizing antibody were likely causes of the recrudescence. Although we were unable to measure T cell responses or drug levels, we feel the most likely possibility for the observed recrudescence is insufficient drug exposure.

## Supplementary Material

1

## Figures and Tables

**Figure 1:**
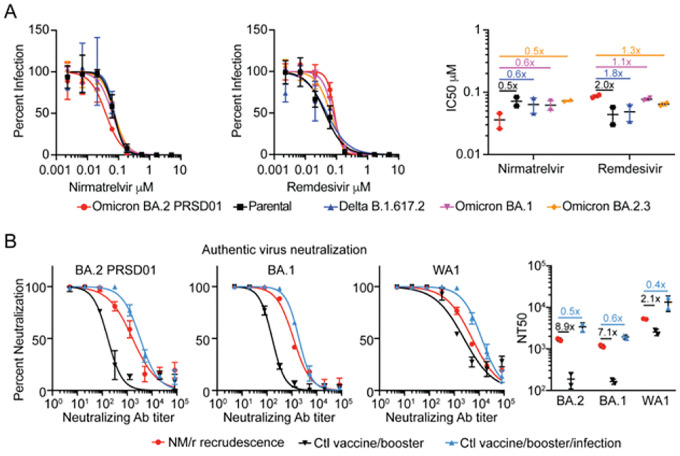
Comparison of antiviral activity and neutralization against BA.2 PRSD01 after NM/r recrudescence. Dose response curves, IC50 and NT50 show the averages ± standard deviation (SD) from 2 independent experiments with 2 biological replicates.
