# Supplementary material for: Virologic and Immunologic Characterization of COVID-19 Recrudescence after Nirmatrelvir/Ritonavir Treatment
Source: Res Sq. 2022 May 18:rs.3.rs-1662783. Preprint. [Version 1] doi: 10.21203/rs.3.rs-1662783/v1 (PMC9128786; doi:10.21203/rs.3.rs-1662783/v1)
Supplement: 1 [file NIHPPRS1662783V1-supplement-1.pdf]

Supplemental Information

Methods:

Study Participants

Nasopharyngeal swabs and plasma samples from participants were obtained under UCSD IRB #200236X and #181624.

SARS-CoV-2 sequencing and sequence analysis

SARS-CoV-2 full genome sequencing was performed using the COVID-19 ARTIC v4 Illumina library construction and sequencing protocol (https://github.com/CDCgov/SARS-CoV-2_Sequencing). Amplicons were generated with the NEBNext VarSkip VSS2b Primer kit (https://github.com/nebiolabs/VarSkip). PCR conditions were 98°C for 30 s, followed by 24 cycles of 98°C for 15 s and 63°C for 5 min, with a final 65°C extension for 5 min. Libraries were generated using the Nextera DNA Flex library preparation kit with Illumina index adaptors and sequenced on a MiSeq instrument (Illumina, San Diego, CA, USA). Samples were sequenced using 300 × 150-bp paired-end reads. Reads were processed with the CLC Genomics Workbench V22 (Qiagen). Briefly the workflow identifies individual SARS-CoV-2 sample variants by first trimming and mapping high quality reads (>20) to the reference genome and then calling variants to generate a full genome consensus for each sample. All consensus sequences were assigned to lineages by Pangolin (PMID: 34527285). Sequences were further aligned to a set of representative SARS-CoV-2 variants using NextAlign (PMID: 29790939). Amino acid variations across all coding regions of the sample isolates as compared to the reference strain BA.2 were interrogated.

Cells and Chemicals

Vero-TMPRSS2 cells (Sekisui XenoTech) were maintained in DMEM plus 10% FBS, 1x Penicillin/Streptomycin, and 0.5 mg/ml Geneticin at 37°C and 5% CO_2. Calu3 cells (ATCC # HTB-55) were maintained in MEM plus 10% FBS, 1x Penicillin/Streptomycin, 1mM sodium pyruvate, and GlutaMAX (Gibco) (Calu-3 media) at 37°C, 5% CO_2. Nirmatrelvir (Selleckchem PF-07321332) and Remdesivir (GS-

5734, AA Blocks, San Diego, CA) were diluted in DMSO to create concentrated stock solutions at 10mM prior to diluting in Calu-3 media for antiviral assays.

SARS-CoV-2 isolation and propagation

TMPRSS2-VeroE6 cells (Sekisui XenoTech) were plated in a 96well plate in DMEM + 2% FBS + Penicillin/Streptomycin. Patient sample stored in viral transport medium at 4°C was twofold serially diluted in DMEM + 2x Antibiotic/Antimycotic (anti/anti) and added to the newly-plated cells. After 7h, inoculum was removed and replaced with DMEM + 2% FBS with 1x anti/anti. When CPE became apparent, supernatant and scraped cells were added to fresh confluent cultures of TMPRSS2-VeroE6. The resulting passage 2 viral supernatant was centrifuged at 1000g for 5 min at 4°C, and aliquots were stored at -80°C. Passage two BA.2 PRSD01 was sequenced and found to have a single non-synonymous mutation at position L3829F (ORF1ab/nsp6 codon 260) and used for experiments. WA1 (NR-52281) and B.1.617.2 (NR-55611) strains were obtained from BEI Resources. BA.1 (GISAID # EPI_ISL_8186377) and BA.2.3 strains were isolated from clinical samples at UC San Diego under IRB #160524. Viruses from clinical samples were isolated on Calu3 cells (BA.2.3) or TMPRSS2-VeroE6 (BA.1, BA.2 PRSD01). All stocks were propagated on TMPRSS2-VeroE6 cells and verified by deep sequencing.

SARS-CoV-2 antiviral assay

Calu3 cells plated 2 days before infection in 96 well plates were pretreated with drugs 60-90min before infection. Cells were infected with a MOI of 0.125 of SARS-CoV-2 variants and incubated 40-44h at 37°C, 5% CO_2. Cells were then fixed with 4.5% formaldehyde for at least 30 min and stained using anti-SARS-CoV-2 nucleocapsid protein (GeneTex, gtx135357) and anti-rabbit AlexaFluor 594 secondary antibody (Thermo Fisher Scientific) with Sytox Green nuclear counterstain. Five images per well were acquired at 10x magnification with an Incucyte S3 imager. Percent infected cells was calculated using Incucyte software tools and best fit curves created using GraphPad Prism 9. Graphs show averages of 2 independent experiments each done using 2 biological replicates.

Authentic SARS-CoV-2 neutralizing antibody assay

Neutralization was determined by focus reduction neutralization test (FRNT). Plasma samples were heat-inactivated at 56°C for 30min, centrifuged at 8000 RPM for 5 min, and then aliquoted and frozen at -80°C until use. Fourfold serial dilutions of plasma samples in DMEM + 1% FBS were incubated with 100-250 focus forming units of authentic SARS-CoV-2 diluted in DMEM for 1h at 37°C. Confluent TMPRSS2-VeroE6 cells in 96 well plates were washed once with PBS then infected with the virus+antibody mixture for 1h with gentle rocking. Inputs were removed, and cells were overlaid with 1% methylcellulose in MEM + 2% FBS and incubated 24h at 37°C. Cells were then fixed and stained for nucleocapsid protein as above, and whole-well images were acquired on an Incucyte S3 imager. Foci were counted using the Incucyte software, and percent neutralization was calculated relative to media-only control wells on each plate. Graphs show averages of 2 independent experiments each done using 2 biological replicates. SARS-CoV-2 neutralization titers were defined as the sample dilution at which a 50% reduction (NT50) in

foci was observed relative to the average of the virus control wells. Best fit curves determining NT_{50} were generated in GraphPad Prism 9. Graphs show averages of 2 independent experiments each done using 2 biological replicates.
